# An Era Ended, But the Legacy Lingers On: A Personal Reflection on Dr. David Baltimore

**DOI:** 10.20411/pai.v10i2.907

**Published:** 2025-11-10

**Authors:** Parameswaran Ramakrishnan

**Affiliations:** 1 Department of Pathology, Case Western Reserve University, Cleveland, Ohio; 2 The Case Comprehensive Cancer Center, Case Western Reserve University, Cleveland, Ohio; 3 Department of Biochemistry, Case Western Reserve University, Cleveland, Ohio; 4 University Hospitals-Cleveland Medical Center, School of Medicine, Case Western Reserve University, Cleveland, Ohio; 5 Louis Stokes Veterans Affairs Medical Center, Cleveland, Ohio

**Keywords:** Reverse Transcriptase, NF-kappaB, Baltimore Classification of Viruses, HIV, Retroviruses, Recombination Activating Gene, Mentorship, Science Policy, Nobel Prize

## Abstract

Dr. David Baltimore's contributions to modern biology span more than six decades and continue to shape the fields of virology, immunology, biochemistry, and molecular biology. Beyond his landmark discoveries—such as reverse transcriptase and NF-κB, as well as the Baltimore classification of viruses—his influence endures through his mentorship, leadership, and the generations of scientists he trained and inspired. In this essay, I recount my journey as his postdoctoral trainee at the California Institute of Technology, offering a personal glimpse into the mind, character, and legacy of a scientist whose approach to thinking, teaching, and living science remains timeless.

## THE POWER OF A SINGLE WORD

When I think of Dr. David Baltimore, the first word that comes to mind is *“think.”* That single word, so deceptively simple, holds the key to unlocking the magic and mystery of science. For David, *thinking* was not merely a cognitive process—it was a philosophy, a lifestyle, and the very foundation of his scientific creativity. In my view, *“think”* to David was what *“imagine”* was to Walt Disney: both words had the power to transport minds into wonderlands of discovery or where the impossible becomes possible.

I was privileged to spend five and a half years in David's “wonderland,” his laboratory at the California Institute of Technology (Caltech), as his postdoctoral trainee. My initial admiration for him and profound interest in his work originated much earlier, at the time I was first introduced to his name through his *Molecular Cell Biology* textbook during my master's studies. At that time, the seed of an almost impossible dream was planted in my mind—learning directly from Dr. Baltimore—one that, remarkably, would later come true.

## FIRST ENCOUNTERS: FROM DREAM TO REALITY

Though I first saw David in person during my Ph.D. at the Weizmann Institute of Science in Israel in 2004, our first one-on-one meeting took place on April 14, 2007—coincidentally, a regional New Year's Day in India, considered auspicious to start new endeavors in life. This was during my dual-purpose visit to Los Angeles to present my research to David, hoping for the chance to join his lab and to attend the 98th American Association for Cancer Research (AACR) annual meeting. That Saturday morning at 9:00 a.m., I walked into his office in the Braun Building at Caltech.

**Figure:**
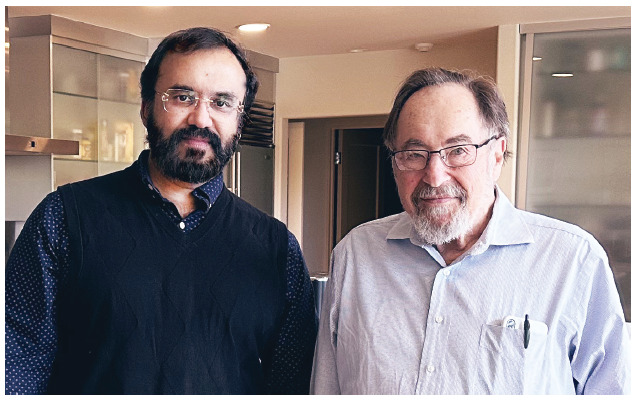
Param Ramakrishnan with David Baltimore. A picture taken at David's house on December 26, 2022.

My heart was racing—part nervousness, part exhilaration—as I realized I was about to sit beside my scientific idol and present my original work, which was a surreal experience.

At the outset, he mentioned he had limited time since he was preparing a keynote lecture for the AACR meeting the following morning. But, as anyone who has interacted with David knows, his curiosity and scientific engagement are boundless, and nothing can stop David's scientific interest. What was meant to be a relatively brief meeting evolved into an intense discussion lasting two hours and forty-five minutes, deeply immersed in biochemistry and molecular biology. Only later did I learn how deeply David valued biochemistry, the discipline that had provided the foundation for his scientific career, back when molecular biology was still in its infancy.

At the end of that meeting, he gently patted my shoulder and said, “I think you will do well in my lab. We need some biochemistry, and I'm happy to offer you a postdoctoral position. Think about it and let me know whether you'd like to accept.” For a few moments, I was speechless. When I finally absorbed what had just happened and managed to respond, I said, “I still can't believe I'm sitting next to you and being offered a position in your lab.” He smiled and replied, “Relax. I'm just another person like you who likes to do science. That's what brings us together.”

That humility, from a man whose discoveries fill chapters of textbooks, whose career spans over six decades with more than 700 publications, and whose influence has shaped the entire field of biology, remains one of the most defining aspects of his character, that allowed him to carry himself with disarming simplicity.

## PATHOGENS AND IMMUNITY—A THEMATIC UMBRELLA

By mere coincidence, specifically for the readers of this journal, most of David's work could be summarized under the theme *“Pathogens and Immunity.”* His groundbreaking discoveries in virology and immunology connected the molecular workings of viruses to the complex choreography of immune responses. His identification of the transcription factor NF-κB—the nuclear factor that binds to the kappa light chain enhancer in B lymphoid cells—elegantly revealed the interface between pathogens and immunity. It deciphered complex molecular and cellular mechanisms regulating infections and the interplay between innate and adaptive immune responses during infections.

## THE POWER OF THOUGHT AND THE PURSUIT OF FOUNDATIONAL SCIENCE

David's career embodies the triumph of logic-driven curiosity. His brilliance was often manifested through simple yet profound reasoning. From his logical deduction and the legendary conclusion—“therefore, there is an enzyme”—that led to the Nobel Prize-winning discovery of reverse transcriptase, which reshaped the central dogma of biology, to his insights into antibody formation and the identification of NF-κB—a molecule that became a cornerstone of modern immunology, his work consistently illuminated the hidden architecture of life. The Baltimore classification of viruses, his discovery of recombination-activating gene (RAG) enzymes mediating antibody and T cell diversity, exploration of protein tyrosine phosphorylation, foundational work on human immunodeficiency virus (HIV) biology, elucidation of NF-κB signaling in inflammation and autoimmunity, and studies on microRNAs—all stand as pillars of molecular science, which makes one often wonder why he never received a second Nobel Prize.

Even as a high school student, his curiosity was evident—he often mentioned he was intrigued by seeing the obese mice at Jackson Laboratories. That early curiosity evolved into a lifelong passion for *in vivo* experimentation and mouse models of disease. Throughout his career, he emphasized that deep biological insights arise from observing living systems in their natural complexity.

## MENTORSHIP: TEACHING INDEPENDENCE THROUGH THINKING

David's mentorship style was both challenging and empowering. The very day he offered me a postdoctoral position, he asked me to draft my proposed research on NF-κB in the format of an NIH R01 grant application. At that time—my second day ever in the United States—I had absolutely no idea what an R01 grant was. Yet, in his own subtle way, David was teaching me to think independently, to plan like a principal investigator. Eventually, my aims were incorporated into a larger NF-κB-focused R01 grant that was funded shortly after I began my work in his lab. I would later learn that I was the last official recruit to join his laboratory, focusing solely on NF-κB research.

On one occasion, he told me something that has stayed with me ever since: “The foundational core content is what endures in science. It doesn't matter what language you think in—what matters is *how deeply* you think. Others can edit your words, but only you can think your thoughts.”

That was quintessential David—an unwavering belief in the power of thought over form. That advice has become a compass for the careers of many of his trainees. David's lectures and writings embodied the same principle—clarity of thought above all. In his lectures, he could distill complex ideas into messages that resonated long after the talk ended. At times, he also entertained his audience impromptu. For example, those who attended his April 15, 2007, AACR talk on Sunday morning will remember the unique way he started his talk and paused after a short while, telling us “that's the Sunday Sermon! Now science,” eliciting laughter from a captivated audience.

David's mastery extended to writing as well. He had an uncanny ability to convey intricate ideas with simplicity and precision in his articles. He always tried to provide critical suggestions to his trainees to improve their research papers, while encouraging them to explore their full potential in scientific writing. While editing one of my manuscripts, he once said, “It's perfectly fine to start a sentence with ‘because'—it prepares the reader for what follows.” On another occasion, he praised my use of the word *thereby*, remarking, “It's a powerful word to express signal transduction—underused in cell signaling papers.” His attention to each word revealed the same meticulous care that defined his experiments. Every word mattered to him—a reflection of his belief that precision in language mirrors precision in thought.

## A SCIENTIST WHO DREW ENERGY FROM THE WORLD

David drew inspiration and energy for his science from the fascination he had with the world around him and his energy seemed inexhaustible. During his 2008 visit to India, he delivered four distinguished lectures in as many days. When I later asked him about the trip, he smiled and said, “It was hectic, but exhilarating. Seeing thousands of students smiling, waving, and attending my lectures reminded me how deeply this country respects science. Their enthusiasm recharged me to do more.” On another occasion, after flying in from London, he came straight to a lab meeting. Seeing him sort through medications, we asked if he was unwell. He laughed, “Not at all—These are just to fix my circadian clock. These drugs are good to do that job.” The next sentence he said was this. “These medicines have years of research behind them, and they work like a charm. I must read before bed what is new on them.” That was quintessential David—endlessly curious, scientifically disciplined, completely unpretentious, blended humor, and grace.

Like life comes out of pictures in fairy tales, David's guidance and aura had the power to make life out of scientific data and images. His guidance often turned chance observations into discoveries. On one occasion, I serendipitously found that a protein previously reported as a loading control in Western blotting showed unexpected changes upon cell stimulation. When I mentioned it to David, he encouraged me to explore it further, saying, “It may be a control for someone else, but perhaps not for you. If your data are solid, follow the lead—see where it takes you.” That open-mindedness defined his lab's spirit—follow the science, not the convention. This openness—to question assumptions and follow evidence wherever it led—defined his lab's culture and was key to its constant evolution.

Over the years, the Baltimore Lab evolved—from virology to molecular biology to immunology—with each transition reflecting David's fearless intellectual curiosity and marking a new chapter in modern biology. His discovery of reverse transcriptase led to the naming of RNA tumor viruses as retroviruses. His subsequent studies extended toward retroviruses and cancer biology, which then extended to HIV, NF-κB signaling in the immune and non-immune cells and expanding frontiers of immunology research. Through every shift, David emphasized the same truth: *foundational science is the wellspring of translational breakthroughs.*

This philosophy guided his co-founding of the Whitehead Institute for Biomedical Research affiliated with Massachusetts Institute of Technology (MIT) in 1982, with entrepreneur Edwin C. Jack Whitehead. The institute's mission—to pursue fundamental biological discovery—mirrored David's lifelong philosophy. Beyond research, David left an indelible imprint on scientific leadership, serving as president of Rockefeller University, Caltech, and the American Association for the Advancement of Science (AAAS). He helped shape policies on recombinant DNA technology, HIV/AIDS, and human genome editing—always advocating for curiosity-driven, ethical science.

## THE SCIENTIST, THE HUMAN, THE HUMORIST

What set David apart was not just his intellect, but his humanity. He treated everyone with equal respect, from first-year undergraduates to fellow Nobel laureates. He possessed a rare ability to adapt to the knowledge level of the person he was speaking with and elevate the conversation to a higher plane of thought. He could tailor his explanations to anyone's level, then lift them to new heights of understanding. Many of his trainees have remarked that he saw their potential before they themselves did, guiding them toward success with gentle precision.

At a lab birthday celebration, he once joked, “One reason I love my profession is that I never feel older—the average age across from me stays the same, and that keeps me young!” Indeed, his energy was boundless, not only for science but also for radiating joy around him, not just in science but in life. He personally hosted the annual lab Christmas party at Caltech's Athenaeum and took great delight in moderating the Yankee Swap gift exchange. He shared his personal joys freely—from the private plane ride piloted by his wife, Dr. Alice Huang, to his fishing adventures in Montana (where he proudly posed for a photo holding a 20-pound trout that he caught), to showcasing his journey through China with a dynamic, slide-driven narrative, and even demonstrating the “Sport” mode of his new Audi on busy U.S. Route 101.

His humor was sharp and spontaneous. Once, the lab arranged for a look-alike from Caltech to surprise him at a weekly lab meeting near his birthday. When David entered and saw his double, he chuckled and said, “Ah, now we can do twice as much science and train twice as many people!” At a dinner, when someone remarked that the sushi was “excellent and reasonably priced,” David dryly replied, “Good sushi and economy don't usually go together.” On another occasion, a visiting faculty member asked David what he felt was the main difference moving from Boston to Southern California. As usual, David's reply was swift, “Aah! (in his unique tone) When I walked out in Boston, people recognized me, and I could not go more than 50 feet without stopping to say hello to someone. But here in California, no one knows me, probably because I have not starred in a movie yet!” Here, one could easily see David's clever evasion of the comparison between the two leading institutes.

Even outside the lab, he engaged deeply with the world. He visited Case Western Reserve University in 2015 to deliver a keynote lecture at the 38th Annual Biomedical Graduate Student Symposium (BGSS). While driving through Cleveland Heights with its large mansions, he remarked, “If a Clevelander ever gets the chance, they should live in one of these storybook houses—they carry history.” Then he astonished me by time traveling to the 1900's and demonstrating his depth of knowledge about local architecture and Cleveland history; he could converse meaningfully with experts in any field—be it science, geography, history, policy, or cuisine. I also recall a lab reunion dinner he hosted for his mentees and lab alumni in Santa Fe in 2012, following a Keystone Symposium on NF-κB. As the waiter proudly started to describe a Bordeaux wine in elaborate detail, David gently took over the conversation, recounting its entire lineage, vineyard origins, the vintners' philosophy, and stories of small French wineries he had personally visited. Everyone at the table watched, smiling—it was classic David: erudite, effortless, endlessly engaging.

## FINAL ENCOUNTERS AND ENDURING WISDOM

My last in-person meeting with David was on December 26, 2022, at his home in Pasadena. We spent two unforgettable hours together. For half of that time, we stood near his koi pond, talking about science, life, and curiosity. He listened intently as I described my independent research and then offered a final piece of advice that remains etched in my heart: “If you believe in something, take it to completion—whether it gives what you expected or something entirely different. Both outcomes are knowledge gained. Leaving something halfway is no different from not doing it at all.” As we talked near the koi pond, I was reminded of a moment years earlier when he had delivered an impromptu lecture on ichthyology to my middle-school-aged, fish enthusiast son, with the same zest he brought to Nobel-level discussions. That was David—a man who approached everything with pure, unfiltered passion, whether it involved fishkeeping or tackling challenges in fundamental biology.

## LEGACY AND CONTINUITY

During my time in his lab—and likely long before—the Baltimore Lab functioned like a miniature research institute unto itself. David often remarked that Caltech's *Division of Biology* had the perfect name, because “real life science should transcend disciplinary boundaries.” My time in his lab was surrounded by projects spanning HIV, microRNAs, T-cell engineering, B-cell biology, myeloid cell differentiation and function, cancer, skin pathology, pregnancy complications, DNA recombination, portable PCR design, and more. Lab meetings felt like multidisciplinary symposia, attended by budding faculty, physician-scientists, and students alike.

Despite his enormous responsibilities, David prioritized his trainees and never distanced himself from daily research life. He attended nearly every lab meeting, and sometimes he would casually walk to a trainee's bench, saying, “We should talk.” Those spontaneous discussions often redirected entire projects, and invariably for the better.

Perhaps his greatest legacy as a mentor lies in the remarkable number of independent scientists he trained—more than 200 faculty members and principal investigators around the world, a feat unlikely to be surpassed. He always talked highly about his mentors at leading institutes in the U.S. and recognized the key role of mentors in shaping the career of trainees. He paid attention to continue the legacy that he received from his mentors to advance his career for his trainees as well. He allowed each postdoc to carry their projects forward into independent careers, ensuring continuity of ideas and expansion of the scientific frontier. In doing so, he passed on not only knowledge but a *tradition* of curiosity, integrity, and fearless inquiry. This fostered independence and continuity. Through his direct trainees, their students, and the generations beyond—his “scientific grandchildren,” as he fondly called them—David's influence and vision will continue to shape the future of modern biology.

## EPILOGUE: THE ETERNAL LEGEND

To borrow from *The Christmas Song*, “Although it's been said many times, many ways,” the legend of Dr. David Baltimore will remain eternal—not only in the annals of science but in the hearts and minds of those privileged to think, learn, and dream in his world. His legacy reminds us that great science begins not with equipment or data, but with the simple act of thinking deeply and daring to wonder. For those interested in hearing more about science from David's perspective, you can read or watch an August 2021 interview conducted by senior editors of *Pathogens and Immunity*.

